# Predicting tingling sensations induced by autonomous sensory meridian response (ASMR) videos based on sound texture statistics: a comparison to pleasant feelings

**DOI:** 10.1098/rstb.2023.0254

**Published:** 2024-08-26

**Authors:** Hiroki Terashima, Kanae Tada, Hirohito M. Kondo

**Affiliations:** ^1^ NTT Communication Science Laboratories, Nippon Telegraph and Telephone, Atsugi, Kanagawa 243–0198, Japan; ^2^ School of Psychology, Chukyo University, Nagoya, Aichi 466–8666, Japan

**Keywords:** autonomous sensory meridian response, tingling sensation, pleasantness, prediction, sound texture statistics, acoustic feature

## Abstract

Sound serves as a potent medium for emotional well-being, with phenomena like the autonomous sensory meridian response (ASMR) showing a unique capacity for inducing relaxation and alleviating stress. This study aimed to understand how tingling sensations (and, for comparison, pleasant feelings) that such videos induce relate to acoustic features, using a broader range of ASMR videos as stimuli. The sound texture statistics and their timing predictive of tingling and pleasantness were identified through L1-regularized linear regression. Tingling was well-predicted (*r* = 0.52), predominantly by the envelope of frequencies near 5 kHz in the 1500 to 750 ms period before the response: stronger tingling was associated with a lower amplitude around the 5 kHz frequency range. This finding was further validated using an independent set of ASMR sounds. The prediction of pleasantness was more challenging (*r* = 0.26), requiring a longer effective time window, threefold that for tingling. These results enhance our understanding of how specific acoustic elements can induce tingling sensations, and how these elements differ from those that induce pleasant feelings. Our findings have potential applications in optimizing ASMR stimuli to improve quality of life and alleviate stress and anxiety, thus expanding the scope of ASMR stimulus production beyond traditional methods.

This article is part of the theme issue ‘Sensing and feeling: an integrative approach to sensory processing and emotional experience’.

## 1. Introduction

We find pleasing sounds increasingly alluring in the contemporary era. Most of us live in urban areas [1], where often the surrounding sounds do not contribute positively to our mental and physical health [2]. Sensory overload in cities can heighten the risk of anxiety, mood disorders and schizophrenia. Thus, discovering sounds that alleviate stress and improve quality of life is a critical societal endeavour. Equally important is understanding the underlying mechanisms of these beneficial effects. Traditional research avenues have mainly explored the impacts of music and speech [3,4]. However, the emerging field of autonomous sensory meridian response (ASMR) [5,6] is gaining increased attention. ASMR is a tingling, static-like distinct sensation across the scalp, back of the neck and upper spine in response to specific audio and visual trigger stimuli, often accompanying positive feelings. ASMR is known for its unique ability to relax and relieve stress, and for causing tingling sensations [5]. This growing area provides crucial insights into the potential uses of sound to improve individual quality of life and mitigate stress and anxiety. Therefore, delving into how sounds, particularly new phenomena like ASMR, evoke beneficial experiences is crucial [7–9]. The ability to manipulate, or even generate, such influential sounds holds potential to bring forth tangible improvements in our quality of life and mental health [10].

Recent interest in ASMR has surged, yet academic insights that could inform better content editing are still in development. The triggering of ASMR relies heavily on the sounds that come before it [6,9]. Although specific ASMR responders comprise 50–60% of people who have watched ASMR videos [11], research often extends to the broader phenomenon of tingling sensations, investigating its related acoustic features and timing [12–14]. Traditional research on pleasantness, a sensation frequently accompanying ASMR, has largely concentrated on music and speech, identifying negative correlations with acoustic features like loudness and sharpness [4]. However, few studies have directly examined the pleasantness that ASMR stimuli induce, and although similarities have been observed, the precise relationship remains ambiguous [15,16]. Since ASMR is often experienced through videos with sounds, and editing audio is usually simpler than editing video, understanding the unique acoustic elements that trigger ASMR could facilitate more accessible content creation. Thus, it is essential to clarify how ASMR and its acoustic features relate, and to draw a comparison with similarly induced feelings of pleasantness.

Previous studies on acoustic features and ASMR (and pleasantness) have several limitations. Koumura *et al.* [14] explored how frisson from listening to ASMR audio links to certain acoustic features—specifically darkness (concentrated power in low frequencies), loudness and compactness—heard about 2 s earlier. However, they did not develop a solid predictive model and only considered a narrow range of audio stimuli, ignoring video stimuli. Pleasantness was assessed merely as an overall impression, not as time-series data, and they found no clear link with frisson. Moreover, the study’s acoustic features were set in advance, had a fixed timescale, and lacked consideration of control and editing ease. Considering these limits, clarifying the temporal link between ASMR video-induced sensory experiences and acoustic features, especially relative to pleasantness, is crucial, so as to create a solid predictive model.

When we consider the possibility of sound editing, sound texture statistics emerge as a promising feature set [17]. Sound texture is a category of sound that is a superposition of many similar acoustic events and is temporally homogeneous, that is, it has properties that remain constant over time (e.g. rainstorms and insect swarms) [17]. As texture-like sounds (e.g. water, fire and wind) have been used to induce feelings of relaxation, investigating and identifying the statistics that characterize specific sound textures will aid understanding of such rich feelings. These statistics are derived from an auditory model that calculates summary statistics closely related to the perception of a sound for a specific time interval. Specifically, the model decomposes sound into frequency channels, extracts envelopes, applies modulation filters and computes sound texture statistics for each stage [17]. Besides their close relationship with perception [18,19], identifying these statistics allows for the synthesis of multiple instances of sound that are perceptually indistinguishable [17,20]. Sound texture statistics are expected to be useful for building predictive models linked to ASMR perception and for offering improved editability.

In this study, we extensively explored tingling sensation and feelings of pleasantness induced by ASMR videos. We concentrated on sound texture statistics [17], known for their contribution to sound editability and strong linkage to auditory perception. Our aim was to build an interpretable model predicting the tingling and pleasantness experienced during ASMR video consumption. Our analysis employed a diverse range of ASMR videos to thoroughly capture the possible temporal variations in tingling and pleasantness. The results revealed that ASMR videos induce unique patterns of tingling sensation and feelings of pleasantness, distinctly different from those induced by conventional nature videos. Upon applying linear predictive models, we found higher prediction performance for tingling than for pleasantness. Additionally, the specific acoustic features contributing to these predictions, and their timing, differed between tingling and pleasantness. We also assessed the predictive power of the model using an independent set of ASMR audio stimuli. This research paves the way to construct distinct predictive models for sensory experiences in ASMR videos, focusing on tingling and pleasantness. It also paves the way for scientifically guided sound editing to optimize the ASMR experience, helping to reduce stress and improve overall well-being.

## 2. Methods

**Figure 1.**
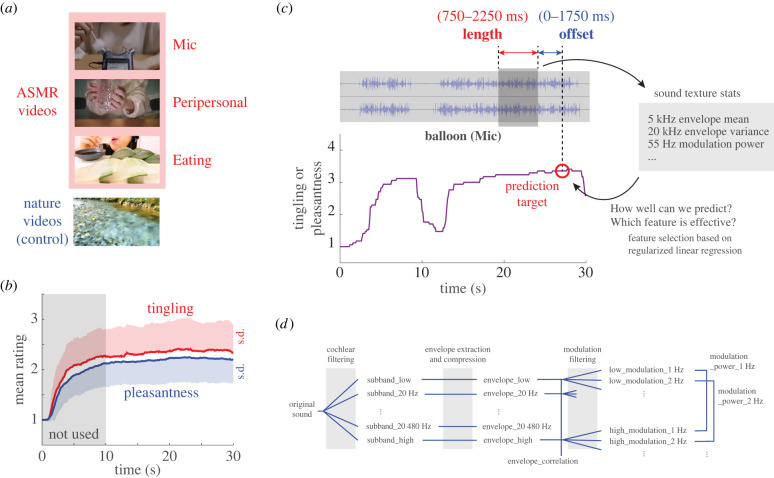
Predictive analysis of tingling and pleasantness in ASMR videos using sound texture statistics. (*a*) Categories of video stimuli. ASMR videos consisted of three categories: Mic, Peripersonal and Eating. Nature videos served as stimuli for comparison. (*b*) Average time series depicting tingling (red) and pleasantness (blue) ratings across all the stimuli of the four categories. Lighter shades of red and blue represent the magnitude of the standard deviations (s.d.) for each. The initial 10 s of unstable ratings (greyed area) were excluded when developing the prediction models. (*c*) Diagrammatic representation of the prediction models used in this research. Ratings (of either tingling or pleasantness) at specific moments in ASMR videos were predicted using the acoustic features in the videos from shortly before those moments. The time series of rating is from the balloon video of the Mic category, averaged across participants. The audio segments used to calculate the acoustic features are determined by two variable parameters: length and offset. We use sound texture statistics as the key acoustic features. Linear regression, grounded in sound texture statistics, assesses prediction performance, while regularization helps to identify specific contributing features. (*d*) Illustrative overview of the auditory model for computing sound texture statistics. The original sound is divided into frequency channels through simulated cochlear filtering, followed by envelope extraction. Subsequently, modulation filtering is applied within each channel and the results for a given modulation frequency are combined by averaging across the envelope frequency channels. (Online version in colour.)

To explore the tingling and pleasantness elicited by ASMR videos, our initial step involved analysing the time series of these experiences during video viewing. Subsequently, we probed how well the time series of sensations could be predicted using acoustic features of ASMR audio, focusing on identifying influential features (figure 1). For acoustic features, we used sound texture statistics, which are intricately linked to perception, based on established auditory models.

### (a) Video stimuli

For this study, we opted for a broader range of stimuli than used in prior research, selecting 15 ASMR videos and five nature videos for comparison (figure 1*a* and table 1; see table S1 in electronic supplementary material for the details). ASMR videos were videos that ASMR artists recorded and edited to maximize ASMR experiences. All videos in this study were recorded by the ASMR artist Hatomugi (https://www.youtube.com/@HatomugiASMR). The ASMR videos were grouped into three categories: ‘Mic’, ‘Peripersonal’ and ‘Eating’, each comprising five videos. To mitigate potential gender bias, we excluded ASMR videos featuring whispering, as most whispering videos are of women whispering. Some videos show the performer in part. Due to limitations in the videos for which permission to use was obtained, all performers in the ASMR videos were female, but, as far as possible, their faces were not shown. Even when they were shown, facial shots were kept to a minimum (only showing the area around the mouth in the Eating videos). Mic videos feature situations in which a performer touches one or more microphones directly (or using an apparatus). Peripersonal videos capture sounds produced close to microphones. Eating videos show a performer slowly chewing common foods, with only the mouth area visible instead of the entire face. These videos focus on the act of eating itself, differentiating them from so-called Mukbang videos [21]. Mukbang often emphasizes the performer’s personality or promotes the food, frequently featuring viewer interaction and entertainment elements. The natural environment videos (category ‘Nature’) functioned as control stimuli to delineate unique properties of ASMR videos, and they were not used in developing predictive models. These nature videos included an exploding flame or flowing water. These nature videos were carefully selected based on criteria ensuring they did not induce ASMR, as confirmed by three raters (including one of the authors), and were specifically chosen to minimize the appearance of individuals within the video frame. Each video was 30 s long, with a resolution of 1280 × 720 pixels, and the audio sampled at 44.1 kHz in stereo.

**Table 1.** ASMR and nature video stimuli.

category	type	description	video name	no. video
Mic	ASMR	touching microphones	Earpick, Massage, Nail, Towel, Balloon	5
Peripersonal	ASMR	surrounding sounds	Gel kneading, Book page turning, Rubbing, Tapping, Glass	5
Eating	ASMR	eating something	Aloe, Fried, Honey, Pickles, Candy	5
Nature	control	natural environments	Walking on leaves, Fire, Fizzing soda, Water, Wind	5
total				20

### (b) Experiment: rating of tingling and pleasantness

#### (i) Participants

We recruited 34 college students (13 males and 21 females; mean ± s.d. age = 21.4 ± 2.6 years). All participants were native Japanese speakers, right-handed, possessing normal hearing, and had normal or corrected-to-normal vision. Participants were randomly divided into two groups: one for the tingling condition and the other for pleasantness (*n* = 17 for each). All participants provided written informed consent following a comprehensive explanation of the study procedures. The sample size was determined prior to data collection, with the possibility of applying an analysis of variance (ANOVA). We were interested in group differences in subjective rating when performing a 2 (tingling versus pleasantness) × 4 (category) mixed-design ANOVA. Sample size (*N* = 34) was based on an *a priori* power analysis with a power of 0.8 (*α*-level = 0.05) to detect a main effect (effect size: Cohen’s *f* = 0.4). We computed sample size using G*Power software (v. 3.1.9.7) [22]. However, we did not perform an ANOVA because we focused on building a predictive model and evaluating it in terms of predictive performance. The validity of the sample size was evidenced by the predictive performance.

#### (ii) Stimuli and procedures

We used twenty 30 s videos, including both ASMR and Nature categories. A 10 s blank screen was displayed between videos. Participants viewed these 20 videos in an order that was randomized for each individual. Before starting, participants performed 1 min practice trials to become familiar with the stimuli and the task procedures. Visual stimuli were displayed on an LCD monitor that had a temporal resolution of 60 Hz. Participants viewed the stimuli at a spatial resolution of 1280 × 720 pixels (visual angle, 16.0 × 9.0°) from a distance of approximately 57 cm. To reduce confounding effects from facial expressions, facial shots were kept to a minimum. Participants were instructed to closely watch the screen, but not to control their blinking. They dichotically listened to the auditory stimuli through Sennheiser HD 599 headphones. Sound pressure levels of the stimuli were measured through an artificial ear (TYPE2015, ACO, Tokyo) and ranged from 54 to 66 dB. Participants were instructed to continuously rate their perceptual experience—either tingling sensation or pleasantness feeling—on a four-point Likert scale: ‘not tingling/pleasant’, ‘weakly tingling/pleasant’, ‘moderately tingling/pleasant’ and ‘strongly tingling/pleasant’. Each stimulus initially started with a rating of 1 (not tingling/pleasant). Responses were collected via four keys on a computer keyboard, sampled at a rate of 1000 Hz. Participants started by holding down the key that indicated a rating of 1. When the intensity of the perceived sensation changed, they switched to and then held down the key corresponding to the new intensity. This process allowed participants to dynamically select and hold down one of the four keys to reflect the subjective intensity of the sensations throughout the experiment. Stimulus presentation and data collection were controlled using a PC with Presentation software (Neurobehavioral Systems, Berkeley, CA, USA).

#### (iii) Preprocessing

We collected the time-series data for the ratings from each participant. These were then averaged, without normalization, to form a collective time series for each stimulus. Figure 1*b* illustrates the averaged time series for both tingling and pleasantness across all videos. The ratings took about 10 s to stabilize, leading us to exclude the initial 10 s from our analysis. Figure 1*c* provides an example of a time series for a single stimulus.

### (c) Acoustic features: sound texture statistics

This study sought acoustic features that are not only closely linked to perception but also easy to manipulate in the time frames under analysis. Accordingly, we adopted sound texture statistics [17], as illustrated in figure 1*d*.

Sound texture statistics are rooted in auditory models and initially break the signal into frequency sub-bands, from which envelopes are extracted. The sound envelope is related to loudness variations over time and is computed using a low-pass filter. Envelopes fluctuating at different speeds are computed using a set of bandpass filters, referred to as modulation filters. Each envelope is thus further partitioned into channels based on modulation frequencies. To enhance interpretability, we limited the number of inter-channel correlations by simple averaging. The modulation power was averaged across identical modulation frequency ranges, and envelope correlations were averaged across all possible combinations. For simplicity, we used arithmetic mean in the results. We additionally tested Fisher’s *r*-to-*z* transform as a method for averaging correlations but found no clear difference in the result of feature selection (although the number of iterations was smaller due to limited computational resource).

These statistics were computed for both the left and right channels. We used their mean and difference as the acoustic features in our predictive model. As a result, our model employed a total of 124 acoustic features, comprising 62 each for average (‘ave’) and difference (‘diff’) categories (table 2). We investigated texture durations in 250 ms increments, ranging from 750 to 2500 ms. To stabilize the computation of circular convolution, a linear fade-in and fade-out were applied to the initial and final 5% of the texture interval. We computed the statistics using the Sound Texture Toolbox (https://mcdermottlab.mit.edu/downloads.html) [17]. For the analyses in this study, we sampled 40 random time points per video.

**Table 2.** Sound texture statistics used for building predictive models.


left-right channels	statistics name	frequency channel	feature label	no. features
average	sub-band mean	low, 20 Hz, 40 Hz, 80 Hz, …, 20 480 Hz, high	ave_sub_mean_{channel}	13
	sub-band variance	low, 20 Hz, 40 Hz, 80 Hz, …, 20 480 Hz, high	ave_sub_var_{channel}	13
	envelope mean	low, 20 Hz, 40 Hz, 80 Hz, …, 20 480 Hz, high	ave_env_mean_{channel}	13
	envelope variance	low, 20 Hz, 40 Hz, 80 Hz, …, 20 480 Hz, high	ave_env_var_{channel}	13
	modulation power	1, 2, 3, 7, …, 221 Hz	ave_mod_pow_{channel}	9
	envelope correlation	—	ave_env_C_mean	1
difference	sub-band mean	low, 20 Hz, 40 Hz, 80 Hz, …, 20 480 Hz, high	diff_sub_mean_{channel}	13
	sub-band variance	low, 20 Hz, 40 Hz, 80 Hz, …, 20 480 Hz, high	diff_sub_var_{channel}	13
	envelope mean	low, 20 Hz, 40 Hz, 80 Hz, …, 20 480 Hz, high	diff_env_mean_{channel}	13
	envelope variance	low, 20 Hz, 40 Hz, 80 Hz, …, 20 480 Hz, high	diff_env_var_{channel}	13
	modulation power	1, 2, 3, 7, …, 221 Hz	diff_mod_pow_{channel}	9
	envelope correlation	—	diff_env_C_mean	1
total				124

### (d) Prediction model for tingling based on acoustic features

To better understand the relationship between tingling and pleasantness and their associated acoustic features, we developed separate models to predict tingling and pleasantness. To enhance the interpretability of the models, linear regression was used, and variable selection was conducted via regularization, allowing for explanations with a reduced set of features (figure 1*c*). The formula for the regression model is as follows:

2.1 $$\begin{array}{rl} y & =\beta_{0}+\beta_{\mathrm{ave}\_\mathrm{sub}\_\mathrm{mean}\_\mathrm{low}}x_{\mathrm{ave}\_\mathrm{sub}\_\mathrm{mean}\_\mathrm{low}}+\beta_{\mathrm{ave}\_\mathrm{sub}\_\mathrm{mean}\_20\;\mathrm{Hz}}x_{\mathrm{ave}\_\mathrm{sub}\_\mathrm{mean}\_20\;\mathrm{Hz}}+\cdots \\ & \;+\beta_{\mathrm{ave}\_\mathrm{env}\_\mathrm{corr}}x_{\mathrm{ave}\_\mathrm{env}\_\mathrm{corr}}\\ & \;+\beta_{\mathrm{diff}\_\mathrm{sub}\_\mathrm{mean}\_\mathrm{low}}x_{\mathrm{diff}\_\mathrm{sub}\_\mathrm{mean}\_\mathrm{low}}+\beta_{\mathrm{diff}\_\mathrm{sub}\_\mathrm{mean}\_20\;\mathrm{Hz}}x_{\mathrm{diff}\_\mathrm{sub}\_\mathrm{mean}\_20\;\mathrm{Hz}}+\cdots \\ & \;+\beta_{\mathrm{diff}\_\mathrm{env}\_\mathrm{corr}}x_{\mathrm{diff}\_\mathrm{env}\_\mathrm{corr}}+\epsilon , \end{array}$$

where *y* is the dependent variable it is desired to predict (either tingling *y*_tingling_ or pleasantness *y*_pleasantness_), *β*_0_ is the intercept, $\beta_{\mathrm{ave}\_\mathrm{sub}\_\mathrm{mean}\_\mathrm{low}},\beta_{\mathrm{ave}\_\mathrm{sub}\_\mathrm{mean}\_\mathrm{low}},\ldots ,\beta_{\mathrm{diff}\_\mathrm{env}\_\mathrm{corr}}$ are the coefficients for the 124 acoustic features $x_{\mathrm{ave}\_\mathrm{sub}\_\mathrm{mean}\_\mathrm{low}},$ $x_{\mathrm{ave}\_\mathrm{sub}\_\mathrm{mean}\_20\;\mathrm{Hz}},\ldots ,x_{\mathrm{diff}\_\mathrm{env}\_\mathrm{corr}}$ and ϵ is the error term.

Figure 1*c* displays the conceptual design of our predictive models. For comparison of prediction performance, we conducted a grid search over two key time-based parameters: offset and length. Each acoustic texture segment, employed for predicting a certain rating, was selected from a preceding time relative to the rating point. The first parameter, the temporal offset, spanned from the end of the acoustic texture segment to the rating point. This was tested at 250 ms intervals, from 0 up to 1750 ms. The second parameter, the length of the acoustic texture segment, was also explored in 250 ms steps, ranging between 750 and 2500 ms. For every parameter combination, 40 random acoustic texture segments were extracted from each video to compile the regression dataset.

For variable selection, we employed least absolute shrinkage and selection operator [23,24], a linear regression method with *L*_1_ norm regularization. We determined the regularization strength, *λ*, through video-stratified 10-fold cross validation (CV). A higher *λ* results in fewer selected variables, aiding in clarity and understanding. To minimize feature count while preserving predictive performance, we adopted the one-standard-error rule [24]. This rule selects the largest *λ* within one standard error of the minimum mean squared error in CV.

#### (i) Evaluation of prediction performance

We evaluated the predictive performance of our models across different temporal offset and segment length combinations (figure 1*c*). Predictive performance was measured using Pearson’s correlation coefficient *r*. It is important to note that the performance metrics from the simple CV process can be overly optimistic due to potential information leakage during selection of the regularization parameter *λ* [25]. To mitigate this, we adopted a nested CV (double CV) approach [26,27]. In our study, the outer CV, designed to assess each model’s overall predictive performance with no information leakage, used a leave-one-video-per-class-out strategy, effectively implementing a category-stratified fivefold CV. We averaged predictive performance across five validation sets to represent the performance of a single outer CV cycle. For selecting *λ* without the outer validation set, the inner CV used a video-stratified 10-fold CV. Our analysis included repeating the nested CV 100 times to average the predictive performance.

#### (ii) Evaluation of feature importance

To assess the significance of each feature selected by our models, we implemented permutation feature importance [28] as our evaluation method. In this technique, a feature’s importance is defined by the extent of predictive performance reduction when that feature’s values are randomly shuffled within the validation set. If a feature is vital to the model, a noticeable decrease in performance is expected. By contrast, if a feature has minimal impact, the change in performance should be minimal. For this analysis, we used the validation sets from the outer CV in our nested CV procedure. We carried out the feature-value shuffling independently on five separate validation occasions, and the average reduction in performance across these instances indicated the importance of the feature within that particular nested CV cycle.

#### (iii) External validation of prediction performance

To further evaluate the predictive performance of our model for tingling, we conducted an additional test with an independent set of ASMR audio stimuli from a previous study [14]. This selection allowed us to assess performance without video stimuli, using a variety of ASMR clips. We applied our best tingling model to these independent set of ASMR audio stimuli. We extracted optimal-length segments from the audio at 1 s intervals, predicting the time-series ratings (averaged across participants) while considering the optimal temporal offset. Consistent with the present study, the first 10 s of the stimuli were not included in the performance evaluation. To align with our study, we linearly transformed the rating range from the earlier study (1–3) to match our range (1–4) for comparison purposes.

## 3. Results

### (a) Distinct patterns of tingling and pleasantness ratings for ASMR videos

**Figure 2.**
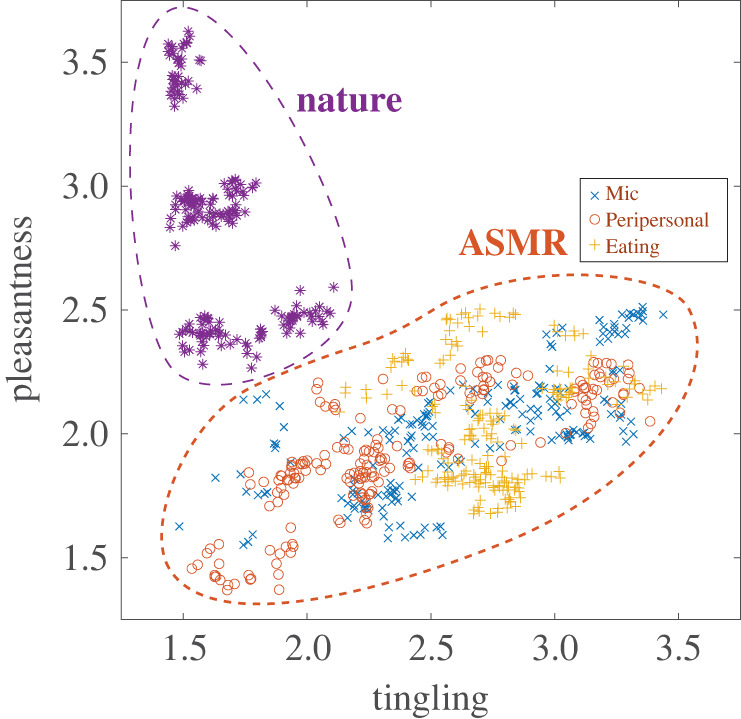
Distinct rating distribution of ASMR videos (red) compared with nature videos (purple). Tingling in ASMR videos is moderately correlated with pleasantness. Typically, ratings for pleasantness were lower than those for tingling. Conversely, in nature videos, ratings for pleasantness were higher than those for tingling. We sampled 40 random time points from each video. (Online version in colour.)

We first explored how tingling and pleasantness are effective in discerning the distinctive features of ASMR videos. Figure 2 illustrates the distribution of tingling and pleasantness. Each mark shows the average score across participants at a randomly chosen point during playback of a video (raw time series before averaging are shown in electronic supplementary material, figures S1 and S2; figure S3 shows correlations between participants). For this figure, we sampled 40 points per video. This visualization highlights two separate clusters: one representing nature videos (purple) and the other ASMR videos (red). Notably, the ASMR cluster often scored higher for tingling, a common emotional response to ASMR content. Conversely, nature videos generally yielded higher pleasantness ratings. This distinction emphasizes the unique nature of ASMR videos compared to nature videos, with tingling and pleasantness being pivotal in highlighting these differences.

### (b) Tingling prediction based on sound texture statistics

#### (i) Establishing the best predictive model

**Figure 3.**
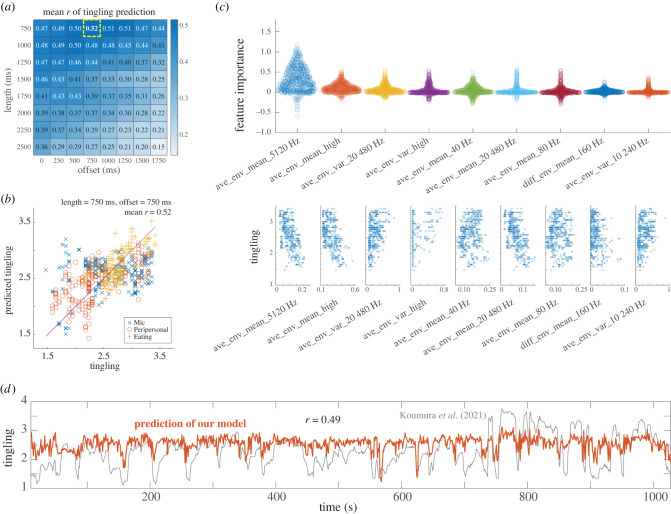
Tingling prediction performance. (*a*) Matrix displaying prediction performance *r* with varied length and offset (average over 100 trials). The best performance occurred at 750 ms for both length and offset (highlighted by yellow dotted lines; *r* = 0.52). (*b*) Distribution of tingling ratings and model predictions, using 750 ms for both length and offset. Distributions from five occasions of validation in the outer CV of a single nested CV are superimposed. (*c*) Distribution of feature importance obtained for each validation occasion during iterations of nested CVs. Upper panel displays features with mean feature importance greater than 0.02 in descending order. Lower panels illustrate the relationship between each feature’s value and tingling rating via scatter plots. (*d*) External validation of prediction performance using different ASMR audio. The red line represents the model’s prediction with both length and offset set at 750 ms. The model achieved a prediction performance of *r* = 0.49; original ratings (ranging from 1 to 3) were linearly transformed to a 1–4 scale for consistency with this study and are plotted in grey.

In constructing a predictive model for tingling based on sound texture statistics, it was essential to identify the appropriate length for sound segments and the length of the time offset. We employed nested CV to robustly estimate the model’s predictive performance and to prevent information leakage. Figure 3*a* illustrates how predictive performance varied with changes in sound segment length and time offset.

The best predictive performance *r* = 0.52 with a low standard deviation (s.d. = 0.08) was achieved using a sound segment length of 750 ms and a time offset of 750 ms. Figure 3*b* displays a scatter plot of tingling ratings versus predicted tingling ratings from a typical single nested CV.

Under these best conditions, the formulated predictive model (incorporating 25 features) is as follows:

3.1 $$\begin{array}{rl} y_{\mathrm{tingling}} & =2.809-5.986x_{\mathrm{ave}\_\mathrm{env}\_\mathrm{mean}\_5120\;\mathrm{Hz}}-8.322x_{\mathrm{ave}\_\mathrm{env}\_\mathrm{mean}\_\mathrm{high}}+0.769x_{\mathrm{ave}\_\mathrm{env}\_\mathrm{var}\_20 480\;\mathrm{Hz}}\\ & \;+0.659x_{\mathrm{ave}\_\mathrm{env}\_\mathrm{var}\_\mathrm{high}}+2.747x_{\mathrm{ave}\_\mathrm{env}\_\mathrm{mean}\_40\;\mathrm{Hz}}+1.501x_{\mathrm{ave}\_\mathrm{env}\_\mathrm{mean}\_80\;\mathrm{Hz}}\\ & \;-3.653x_{\mathrm{diff}\_\mathrm{env}\_\mathrm{mean}\_160\;\mathrm{Hz}}+(\mathrm{other}18\mathrm{termsoffeatureimportance}<0.02). \end{array}$$

#### (ii) Investigating feature importance

To assess the contribution of each variable to the predictions of our model (equation (3.1)), we used the permutation feature importance technique. Figure 3*c* presents the features that showed high importance during the iterations of nested CV, including the corresponding scatter plots. Features linked to differences between left and right channels were less frequently selected and demonstrated lower importance than averages. Negative correlations were found between tingling and the mean envelope of higher frequency channels (5120 Hz, high, 20 480 Hz). Conversely, positive correlations were observed for the variance of the envelope of the higher frequency channels (20 480 Hz, high, 10 240 Hz) and the mean of the lower frequency envelope (40 Hz, 80 Hz).

#### (iii) Assessing validity of the predictive model

To assess the validity and generalizability of our predictive model for tingling, we conducted further tests using independent ASMR stimuli. For this validation, we used ASMR audio and corresponding ratings [14], providing an opportunity to test the model’s adaptability in conditions differing from those with videos. The time-series predictions are illustrated in figure 3*d*. When we excluded the initial 10 s, following our model’s construction approach, the model demonstrated a correlation of *r* = 0.49.

### (c) Pleasantness prediction based on sound texture statistics

#### (i) Establishing the best predictive model for pleasantness

**Figure 4.**
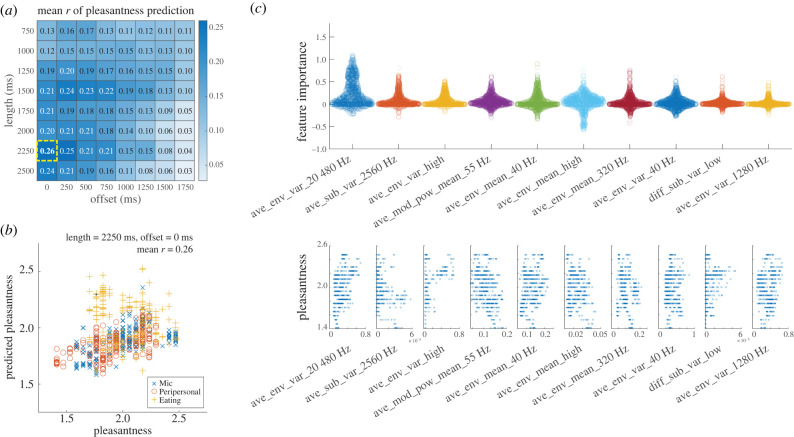
Pleasantness prediction performance. (*a*) Prediction performance matrix *r* with varying segment length and time offset (average over 100 trials). The best performance was observed for a length of 2250 ms and an offset of 0 ms (highlighted by yellow dotted lines; *r* = 0.26). (*b*) Distribution of pleasantness and predicted ratings, using a length of 2250 ms and an offset of 0 ms. Distributions from five occasions of validation in the outer CV of a single nested CV are superimposed. (*c*) Overview of feature importance obtained for each validation occasion during iterations of nested CVs. Upper panel displays features with mean feature importance greater than 0.02, ordered by decreasing importance. Lower panels illustrate the relationship between each feature’s value and the pleasantness rating via scatter plots. (Online version in colour.)

Following the same approach as for tingling, we also constructed a predictive model for pleasantness. Figure 4*a* demonstrates the variations in predictive performance as both the sound segment length and time offset were altered.

The best predictive performance was *r* = 0.26 (s.d. = 0.13). This was achieved with a sound segment length of 2250 ms and a time offset of 0 ms (figure 4*a*). It is noteworthy that the predictive performance of the model for pleasantness was lower than that of the model for tingling. Figure 4*b* is a scatter plot showing the distribution of predicted pleasantness against actual ratings in a typical single run of nested CV.

The best predictive model for pleasantness (66 features) was established as follows:

3.2 $$\begin{array}{rl} y_{\;\mathrm{pleasantness}} & =3.019+0.913x_{\mathrm{ave}\_\mathrm{env}\_\mathrm{var}\_20 480\;\mathrm{Hz}}+-4815.474x_{\mathrm{ave}\_\mathrm{sub}\_\mathrm{var}\_2560\;\mathrm{Hz}}+0.350x_{\mathrm{ave}\_\mathrm{env}\_\mathrm{var}\_\mathrm{high}}\\ & \;-2.177x_{\mathrm{ave}\_\mathrm{mod}\_\mathrm{pow}\_\mathrm{mean}\_55\mathrm{Hz}}+2.017x_{\mathrm{ave}\_\mathrm{env}\_\mathrm{mean}\_40\;\mathrm{Hz}}-11.096x_{\mathrm{ave}\_\mathrm{env}\_\mathrm{mean}\_\mathrm{high}}\\ & \;-0.647x_{\mathrm{ave}\_\mathrm{env}\_\mathrm{mean}\_320\;\mathrm{Hz}}-0.838x_{\mathrm{ave}\_\mathrm{env}\_\mathrm{var}\_40\;\mathrm{Hz}}+3099.744x_{\mathrm{diff}\_\mathrm{sub}\_\mathrm{var}\_\mathrm{low}}\\ & \;+0.634x_{\mathrm{ave}\_\mathrm{env}\_\mathrm{var}\_1280\;\mathrm{Hz}}+(\mathrm{other}56\mathrm{termsoffeatureimportance}<0.02). \end{array}$$

#### (ii) Investigating feature importance

We also assessed the importance of individual features in the pleasantness model (equation (3.2)) using the permutation feature importance technique. Figure 4*c* illustrates the features that demonstrated relatively high importance during the nested CV iterations, along with their respective scatter plots.

The features that showed relatively high importance were the envelope statistics of the higher frequency bands (variance in 20 480 Hz; mean and variance in the highest frequency channel). At lower frequencies, the importance of the 40 Hz envelope and the 55 Hz modulation was high; the features with high importance were predominantly left-right averages, akin to the tingling model. However, it is important to note that the overall magnitude of feature importance was generally lower than for the tingling model, and distinct patterns in the scatter plots were less evident.

**Figure 5.**
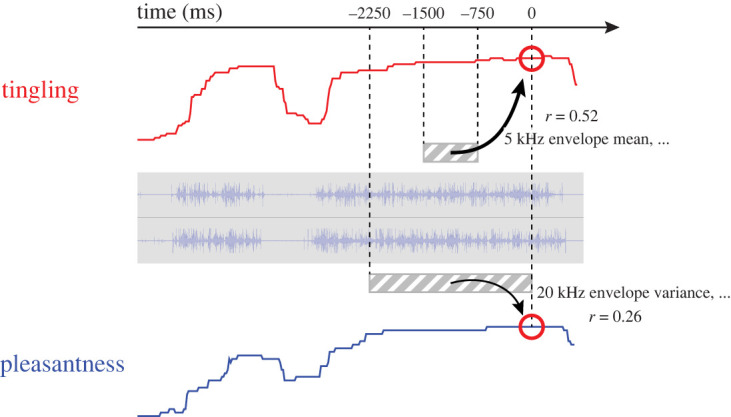
Summary of the key findings of this study. In ASMR videos, tingling at a specific moment (red circle; 0 ms) was well predicted using sound texture statistics spanning from −1500 ms to −750 ms (grey shaded area), achieving a correlation coefficient of *r* = 0.52. A notable predictor was the left-right average of the 5 kHz envelope mean. Predicting pleasantness required a broader time window of sound texture statistics, extending from −2250 ms to 0 ms, yielding a lower prediction performance (*r* = 0.26). (Online version in colour.)

Figure 5 summarizes the main results of this study. The rating of tingling at a given moment (red circle at 0 ms) was well-predicted using sound texture statistics from −1500 ms to −750 ms (grey shaded area), with a particular emphasis on the 5 kHz envelope mean. By contrast, when applying similar methods for predicting pleasantness, the prediction performance was lower than for the tingling model. Effective features were high-frequency envelope variance from −2250 ms to 0 ms (the point of rating), indicating a reliance on a longer timescale than for tingling.

## 4. Discussion

We developed predictive models for tingling and pleasantness, two key emotional experiences induced by ASMR videos, using sound texture statistics (figure 1*c*). Key insights from our study are:

— A clear distinction between ASMR and nature videos, with tingling and pleasantness as defining characteristics (figure 2).
— The best prediction of tingling was achieved using a texture length of 750 ms and time offset of 750 ms, resulting in a predictive performance of *r* = 0.52 (figure 3*a*,*b*).
— Left-right averages of high- and low-frequency envelopes played a more significant role in tingling prediction than did left-right differences (figure 3*c*).
— The tingling model generalized well to an independent set of ASMR audios (figure 3*d*).

For pleasantness, a greater length of 2250 ms without time offset was required, and predictive performance was lower (*r* = 0.26; figure 4*a*,*b*). Fewer features exhibited a clear relationship with pleasantness (figure 4*c*).

The acoustic features linked to ASMR discovered in this study largely align with previous research [14], while providing additional insights and novel findings, particularly in the frequency domain. The previous study [14] indicated that frisson (tingling) induced by ASMR audios was positively correlated with amplitude and bandwidth, and negatively with frequency-centroid (all left-right averages). Our model supports this by showing a positive correlation between the mean of the low-frequency envelope and tingling. Essentially, our findings suggest that more resonant sounds, similar to the rumble of bass tones, are more likely to trigger ASMR, underlining the importance of volume of low-frequency sounds. At first glance, the positive correlation with amplitude may seem to conflict with the negative impact of high-frequency envelopes in our tingling model (equation (3.1)). This apparent contradiction is resolved when the positive correlation of low-frequency envelopes (40 and 80 Hz) is also considered (figure 3*c*), indicating that the amplitude effects shown in the previous study primarily stem from low frequencies. This implies that the correlation between amplitude and tingling is stronger for low-frequency sounds. Moreover, our model reveals that high-frequency sounds do not always diminish ASMR; rather, the variances of their envelopes were positively correlated with tingling (figure 3*c*).

Pleasantness might be considered as a higher-order concept less directly affected by low-level acoustic features [29,30]. The failure to consistently narrow down the features that are helpful for prediction of pleasantness (equation (3.2)) supports this view.

In our investigation of best time parameters for prediction, we discovered that a time offset of 750 ms was most effective for predicting tingling. A total duration of 1500 ms including a texture length of 750 ms is comparable to the approximately 2 s time delay found in the previous study on ASMR audio frisson [14]. Such correspondence indicates that the effective timescales for triggering tingling are consistent, irrespective of the presence of video components or the choice of acoustic features.

Notably, the best timescales for predictions differed between tingling and pleasantness (summarized in figure 5). For predicting pleasantness, a longer texture length of 2250 ms was required. This variance in the best timescales suggests that the mechanisms underlying tingling and pleasantness may function on different temporal dimensions. Indeed, crisp-like transient sounds have been noted as a representative trigger of ASMR [5].

Concerns that our models might merely be distinguishing between different categories of stimuli are unfounded. Although our initial analysis incorporated nature videos as a distinct category, they were not used in the subsequent model development. The ASMR videos demonstrated considerable diversity in their ratings, with categories like Mic, Peripersonal and Eating showing substantial overlap in their rating distributions (figure 2). Importantly, one point that cannot be explained by mere category prediction is that our model can predict the moment-to-moment fluctuation of rating, not averaged feelings for the entire video. Thus, these models go beyond simple category classification.

### (a) Limitations

The primary limitation of this study arises from its focus on sound texture statistics from a particular set of stimuli for prediction. The demonstrated predictive performance might represent a lower bound, as other acoustic features could potentially improve the models. For example, it might be beneficial to include the feature set used in the previous research [14] or binaural hearing features like interaural time differences (ITDs) and interaural level differences (ILDs). However, these features present more complexity in terms of control and unclear causal relationships. Despite this, our model showed a relatively minor role for ILD-related features (e.g. diff_mean_envelope). Another area for future exploration concerns how sound editing, based on our predictive models, can change the degree of feelings induced by ASMR videos. Subsequent research should evaluate the robustness of our model for tingling when sound texture statistics (and sounds) are altered through editing techniques. Although our validation with audio-only stimuli suggests that visual components might have a limited impact on ASMR experiences, the interplay between audio and visual stimuli in ASMR is still a relatively unexplored area. Considering this, a valuable future direction could involve experiments focusing specifically on visual elements. Future studies could test the effects of visual edits and provide insights into how visual changes influence the overall ASMR experience.

Besides the limitations already noted, the study faces challenges in generalization, primarily due to the limited range of stimuli examined. Future research focusing on other ASMR aspects, rather than tingling experience, will require careful methodological choices, including pre-selection of ASMR responders [11,31] and the inclusion of whisper videos [32,33]. It it noteworthy that the Japanese term ‘zoku-zoku’ was used to describe the tingling sensation in the present study (and frisson in the previous study [14]), raising potential issues with cross-cultural and linguistic generalizability of our findings. Additionally, even though the videos did not, for the most part, show the face, the exclusive use of stimuli from a single female ASMR artist limits the broad applicability of our results. However, this limitation was somewhat mitigated by our validation using an independent set of stimuli.

### (b) Contributions in a broader context

An important strength of this study is the novel approach to create comparable models of tingling sensation and pleasant feeling, using the same ASMR video stimuli. It also contributes to establishing a new quantitative benchmark for empirical research in the developing field of ASMR. Our results show that tingling can be predicted more easily, benefits from a shorter predictive timescale, and depends on different acoustic features than pleasantness.

This study also extends the body of knowledge on sound texture statistics by showing that there are other useful scenarios besides their originally proposed usages [17]. To the best of our knowledge, this is the first instance of sound texture statistics being applied directly to predict tingling or pleasantness. The statistics can be seen as nonlinear features linked to the auditory system, a set of features specialized in the auditory system compared with general nonlinear terms (e.g. those used in growth curve modelling). We used sound texture statistics as a kind of auditory nonlinear features rather than the features that characterizes sound textures in the original sense [17]. This suggests that sound texture statistics can be valuable in a broader spectrum of auditory perception research domains than those that use the statistics in the original sense and with the timescale originally proposed to be effective.

An additional benefit is the application to sound editing. Our results are consistent with previous findings indicating that human-produced sounds more effectively elicit ASMR than nature-produced sounds [34] (figure 2). As highlighted in McDermott & Simoncelli [17], sound texture statistics permit targeted modifications through sound editing techniques. This approach paves the way for innovative ASMR stimuli production, advancing beyond traditional methods [6]. Such progress may have practical applications in enhancing individual well-being and quality of life, as well as in the targeted alleviation of stress and anxiety [7–9,35,36].

## Data Availability

The datasets supporting this article (behavioural data and MATLAB sample code) have been uploaded as part of the supplementary material. To run the sample code, the Sound Texture Toolbox is necessary (https://mcdermottlab.mit.edu/downloads.html). Due to restrictions on the distribution of ASMR videos, for validation purposes, please reach out to the authors or the artist directly. The data are provided in electronic supplementary material [37].
